# HOX-7 suppresses body weight gain and adipogenesis-related gene expression in high-fat-diet-induced obese mice

**DOI:** 10.1186/1472-6882-14-505

**Published:** 2014-12-17

**Authors:** Heon-Myung Lee, Hong-Kun Rim, Jong-Hwan Seo, Yoon-Bum Kook, Sung-Kew Kim, Chang-Hyun Oh, Kyung Ho Yoo, Jong-Sik Jin, Hyo-Jin An

**Affiliations:** Department of Pharmacology, College of Oriental Medicine, Sangji University, Wonju-si, Gangwon-do 220-702 Republic of Korea; Department of Presctiption, College of Oriental Medicine, Sangji University, Wonju-si, Gangwon-do 220-702 Republic of Korea; Center for Biomaterials, Korea Institute of Science and Technology (KIST), PO Box 131, Cheongryang, Seoul, 130-650 Republic of Korea; Chemical Kinomics Research Center, Korea Institute of Science and Technology (KIST), PO Box 131, Cheongryang, Seoul 130-650 Republic of Korea; Department of Oriental Medicine Resources, College of Environmental & Bioresources Sciences, Chonbuk National University, Jeonju, Jeonbuk Republic of Korea

**Keywords:** C/EBPα, Mice, Obesity, PPARγ, SREBP1c, Traditional herbal medicine

## Abstract

**Background:**

HOX-7 is a newly developed dietary formula composed of traditional oriental herbal medicines. The formula was developed with the aim of improving weight control. We investigated the anti-obesity effect of HOX-7 on high-fat-diet (HFD)-induced obesity in C57BL/6 mice.

**Methods:**

The mice were divided into four groups and were fed a normal diet (ND), HFD, or HFD with oral administration of HOX-7 at 100 or 200 mg/kg/day for 12 weeks. Body and fat weight, histological changes of fat tissue, and the expression of key adipogenic transcription factors were investigated.

**Results:**

The body weight of mice fed the HFD with HOX-7 was significantly decreased compared to the HFD group. There were no obvious differences in weekly food intake among the 4 groups. The weight of the epididymal and total fat pads was reduced in mice fed the HFD with HOX-7. Treatment with HOX-7 also substantially attenuated the expression of key adipogenic transcription factors, including peroxisome proliferatoractivated receptor γ, CCAAT/enhancer binding protein α, sterol regulatory element binding protein 1c, adipocyte P2, liver X receptor, and lipoprotein lipase in the epididymal adipose tissue.

**Conclusion:**

Overall, this study highlighted the anti-obesity effects of HOX-7, a finding that could contribute to the development of natural anti-obesity herbal medicines.

## Background

Obesity is one of the most prevalent metabolic diseases in the world and constitutes a serious threat in both industrialized and developing countries [1]. It is connected with the genesis or development of various diseases, such as hypertension, cardiac arrhythmia, constipation, headache, steatorrhea, type 2 diabetes mellitus, and cancer [2, 3]. The process of gaining fat involves increased adipogenesis accompanied by adipocyte differentiation. This differentiation is regulated by a highly organized cascade involving numerous transcription factors [4]. Among them, peroxisome proliferator-activated receptor γ (PPARγ), CCAAT/enhancer binding protein α (C/EBPα), and sterol regulatory element binding protein 1c (SREBP1c) are the major transcriptional genes involved in adipogenesis [4, 5].

To target obesity, current pharmacotherapy strategies are grouped into two categories [6]. The first category is to medicate people with obesity with drugs, including suppressants and anorexics, that can suppress food intake by regulating the central nervous system. The second category involves drugs that inhibit the absorption of specific nutrients in food [7]. However, these drugs, of which there are two varieties, can have undesirable side effects, such as hypertension, cardiac arrhythmia, constipation, and headache [8].

Because of the unavoidable side effects of the currently available anti-obesity drugs, the development of herbal medicinal products to treat obesity has become a global focus and there are many studies concerning natural medicines that might have beneficial effects in obese patients. Some natural compounds, plants and formulas, such as berberine, prunetin, aster glehni, and taeumjowi-tang have proven anti-obesity effects [9–12]. As well as these studies, there is a lot of research in progress globally to develop effective and safe natural anti-obesity drugs.

HOX-7 is a newly combined formula for treating obesity, composed of seven herbs (Coicis semen, Nelumbo nucifera, Saliconia herbacea, Polygoni Multiflori Radix, Raphani semen, Piperis Longi Fructus, and Atractylodis Rhizoma). Coicis semen is an adlay seed and was reported to have anti-obesity effects in humans [13]. Nelumbo nucifera has also been reported to show anti-obesity and hypolipidemic effects and decrese the expression of PPARγ [14]. Recent research on Saliconia herbacea revealed its antioxidant and anti-proliferative properties [15]. In addition, Saliconia herbacea has also been reported to have an adipogenesis inhibition effect [16]. Polygoni Multiflori Radix has been used in the treatment of obesity and hyperlipidemia in East Asian countries for centuries, and a recent study demonstrated its effects in obesity [17]. Raphani Semen, commonly known as radish seed, is used as a traditional medicine to treat constipation, chronic tracheitis, and hypertension [18]. Furthermore, Raphani Semen, which has a high linolenic acid content, could have effects that reduce the risk of cardiovascular disease and was revealed to have anti-oxidative activity [19]. Piperis Longi Fructus was verified for its anti-diabetic and anti-hyperlipidemic effects [20]. Atractylodis Rhizoma was discovered to have an anti-inflammatory effect [21] and anti-obesity effect in 3T3-L1 cells and animal model [22]. Based on these studies, we examined the anti-obesity effects of HOX-7 in high-fat-diet (HFD)-induced obese mice.

## Methods

### Reagents

Coicis semen, Nelumbo nucifera, Saliconia herbacea, Polygoni Multiflori Radix, Raphani Semen, Piperis Longi Fructus, and Atractylodis Rhizoma were purchased from Omniherb Co. Ltd (Daegu, Republic of Korea). The normal diet (ND) and HFD were obtained from Research Diets (New Brunswick, NJ, USA). PPARγ, C/EBPα, SREBP1c, and β-actin monoclonal antibodies were purchased from Santa Cruz Biotechnology (Santa Cruz, CA, USA). The other reagents were purchased from Sigma-Aldrich (St. Louis, MO, USA).

### Preparation of HOX-7 extract

HOX-7 is composed of Coicis semen (260 g), Nelumbo nucifera (416 g), Saliconia herbacea (91 g), Polygoni Multiflori Radix (130 g), Raphani Semen (143 g), Piperis Longi Fructus (52 g), and Atractylodis Rhizoma (208 g). The dried herbs were refluxed with 70% ethanol for 3 h at 80°C. The extract was filtered and refluxed again under the same conditions. After filtration, the extract was completely dried using a freeze-dryer to obtain a solid ethanol extract. The yield was 17% (17 g per 100 g of total materials)

### Animal experiments

Male C57BL/6 J mice (3 – 4 weeks) were purchased from Daehan Biolink (DaeJeon, Republic of Korea). Mice were maintained (4 mice/cage) under a 12 h light/dark cycle, at 22 ± 2°C, and a relative humidity of 55 ± 10%, under conditions that followed the National Institutes of Health guidelines and were approved by the Ethical Committee for Animal Care and the Use of Laboratory Animals, Sangji University (reg. no. 2014–3). Mice were given the diet and water ad libitum. After 1 week of acclimation, 32 mice were divided randomly and equally into 4 groups (*n* = 8 per group); the ND group, HFD group, and HFD with HOX-7 at 100 or 200 mg/kg (H100 or H200 respectively). ND group were fed a commercial standard chow diet and HFD group were fed the HFD for 12 weeks. HOX-7 was dissolved in DW and, orally and daily administrated for 12 weeks with HFD. Food intake and body weight were recorded every week. At the end of the experimental period, the mice were fasted overnight. The next day, animals were anesthetized with zoletil (Virbac, Carros Cedex, France) and their visceral fat pads were excised, weighed immediately, and stored at -80°C.

### Histological analysis

Representative epididymal adipose tissues from each group were fixed with 4% paraform aldehyde and embedded in paraffin. Sections of adipose tissue were cut and stained with hematoxylin and eosin (H&E) for analysis of adipocyte surface area and diameter. The images of the stained slides were observed using a SZX10 microscope (Olympus, Seoul, Republic of Korea) and photographed. Digital images were taken from each slide (5 per group), and adipocyte diameters were measured using Image J software.

### Quantitative Real-time polymerase chain reaction (PCR) analysis

Total RNA was isolated from the homogenized epididymal adipose tissues using TRIzol Reagent (Life Technologies, NY, USA), according to the manufacturer’s instructions. The total RNA was then quantified using an Epoch micro-volume spectrophotometer system (BioTek Instruments Inc, VT, USA). Synthesis of cDNA was carried out with quantified equal amounts of total RNA using a high-capacity cDNA reverse transcription kit (Applied Biosystems, CA, USA). The program was set for 60 min of initiation at 45°C, followed by 5 min of incubation at 95°C, and then maintained at 4°C. A Step One Plus® Real-time PCR system with an SYBR Green Master Mix (Applied Biosystems) and primers (Bioneer, Seoul, Republic of Korea) were used to perform a real-time PCR. All primer sequences are shown in Table 1. The steps were as follows: 10 min at 95°C, 40 cycles of 15 s at 95°C, 45 s at 58°C, a final melting curve of 15 s at 95°C, 1 min at 60°C, and 15 s at 95°C. Fold changes of gene expression were calculated using the comparative threshold cycle (Ct) method (Applied Biosystems). Each value was normalized for the initial control, glyceraldehydes-3-phosphate dehydrogenase (GAPDH).

**Table 1:** **Primer sequences**

Gene name	Forward primers (5′-3′)	Backward primers (5′-3′)
**PPARγ**	TTCGGAATCAGCTCTGTGGA	CCATTGGGTCAGCTCTTGTG
**C/EBPα**	AAACAACGCAACGTGGAGAC	ACCAAGGAGCTCTCAGGCAG
**SREBP1c**	ATCGCAAACAAGCTGACCTG	AGATCCAGGTTTGAGGTGGG
**LXR**	TCCTACACGAGGATCAAGCG	AGTCGCAATGCAAAGACCTG
**LPL**	AGGACCCCTGAAGACACAGC	TTGGGCACCCAACTCTCATA
**aP2**	AGCATCATAACCCTAGATGG	GAAGTCACGCCTTTCATAAC

### Western blot analysis

The epididymal tissues dissected from the experimental animals were homogenized in a commercial lysis buffer PRO-PREP® (Intron Biotechnology Inc., Gyeongi-do, Republic of Korea) and centrifuged at 13000 rpm (4°C) for 5 min. Subsequently, the supernatant was transferred to a fresh 1.5 ml tube. The quantification of the protein was carried out using Bio-Rad protein assay reagent. Each protein sample was loaded on Tris-glycine SDS-polyacrylamide gels, followed by transfer to polyvinylidene difluoride (PVDF) membranes (Millipore, MA, USA). The membranes were then blocked with 5% skim milk in Tris-buffered saline containing 0.1% Tween 20 (TBST) at room temperature for 1 h and incubated at 4°C overnight with 1:1000 dilutions of the primary antibodies. The following day, the membranes were washed with TTBS 3 times for 10 min each and reacted with 1:2500 dilutions of the secondary antibodies for 2 h at room temperature. After reaction, immunoreactive protein bands were visualized using enhanced chemiluminescence (Santa Cruz Biotechnology). Bio-rad Quantity One® Software was used for the densitometric analysis.

### Statistical analysis

All the values reported have been expressed as the mean ± SE. Data were analyzed using one-way analysis of variance (ANOVA) with Dunnett’s test. Statistical analysis was performed using GraphPad Prism (version 5) and *P* < 0.05 was accepted as statistically significant.

## Results

### Effects of HOX-7 on body weight and total fat mass in HFD-induced obese mice

**Figure 1:**
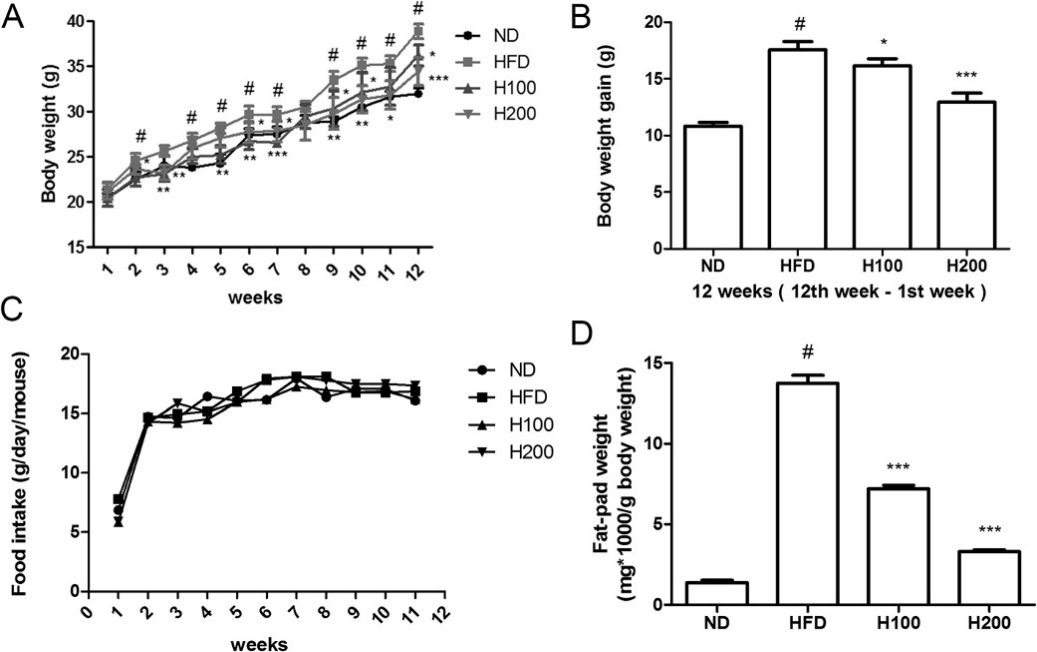
**Effects of HOX-7 on body weight gain, fat-pads weights in HFD mice.** Mice were fed ND or HFD for 12 weeks in the presence or absence of HOX-7. **(A)** Total body (g). **(B)** Body weight gain (g). **(C)** Food intake. **(D)** Visceral fat-pad weight. ND: Normal diet group; HFD: High fat diet group; H100: HOX-7 (100 mg/kg/day, po) treated with HFD group; H200: HOX-7 (200 mg/kg/day, po) treated with HFD group. The values are represented as mean ± SE (n = 8). ^#^ *P* < 0.05 versus ND group. *P < 0.05, **P < 0.01, and ***P < 0.001 versus HFD group.

Figure 1 indicates the changes in body weight and the visceral fat pads, both of which were obviously greater in the HFD group than in the ND group. However, the weights in the H100 and H200 groups were less than those in the HFD group (Figure 1A). Moreover, although there was no significant difference in food intake, the weight gain showed certain differences between the HFD group and HOX-7 groups (Figure 1B and C). Changes in the weight of the visceral fat pads, including the epididymal, retroperitoneal, and mesenteric fat pads, were in line with body weight changes (Figure 1D). The HFD group fats were more highly induced than any other group, whereas the HOX-7 groups showed evident suppression of fat production.

### Effect of HOX-7 on the accumulation of lipid droplets in epididymal adipose tissue

**Figure 2:**
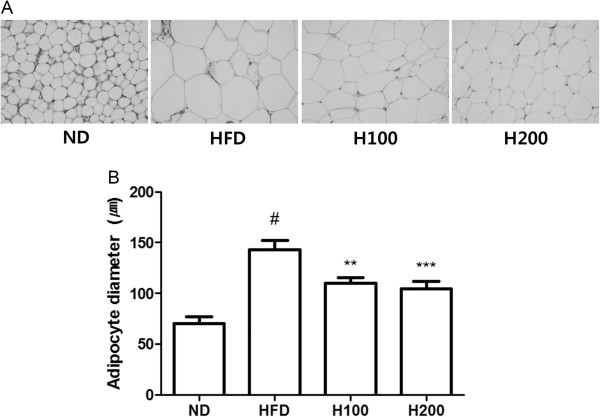
**Effect of HOX-7 on accumulation of lipid droplets in epididymal adipose tissue. (A)** Representative histological images of the epididymal adipose tissue were assessed by H&E staining and examined using a light microscope; magnification: ×400. **(B)** Adipocyte diameter. ND: Normal diet group; HFD: High fat diet group; H100: HOX-7 (100 mg/kg/day, po) treated with HFD group; H200: HOX-7 (200 mg/kg/day, po) treated with HFD group. ^#^ *P* < 0.05 versus ND group. **P < 0.01, and ***P < 0.001 versus HFD group.

To determine whether the observed decrease in body and visceral fat weight was due to reduced accumulation of fat, we stained representative adipose tissues with H&E. As shown in Figure 2, enlargement of adipocytes in the visceral adipose tissue of mice in the HFD group was clearer than that the ND group. In addition, it was revealed that lipid accumulation in the visceral adipose tissue was definitely suppressed in the H100 and H200 groups compared with the HFD group (Figure 2A). Because fatty degeneration in adipose tissues is easy to identify, we compared the diameter of lipid droplets in the adipose tissues. As shown in Figure 2B, administration of HOX-7 significantly decreased the diameter of the lipid droplets.

### Effects of HOX-7 on the expression of adipocyte-specific mRNAs

**Figure 3:**
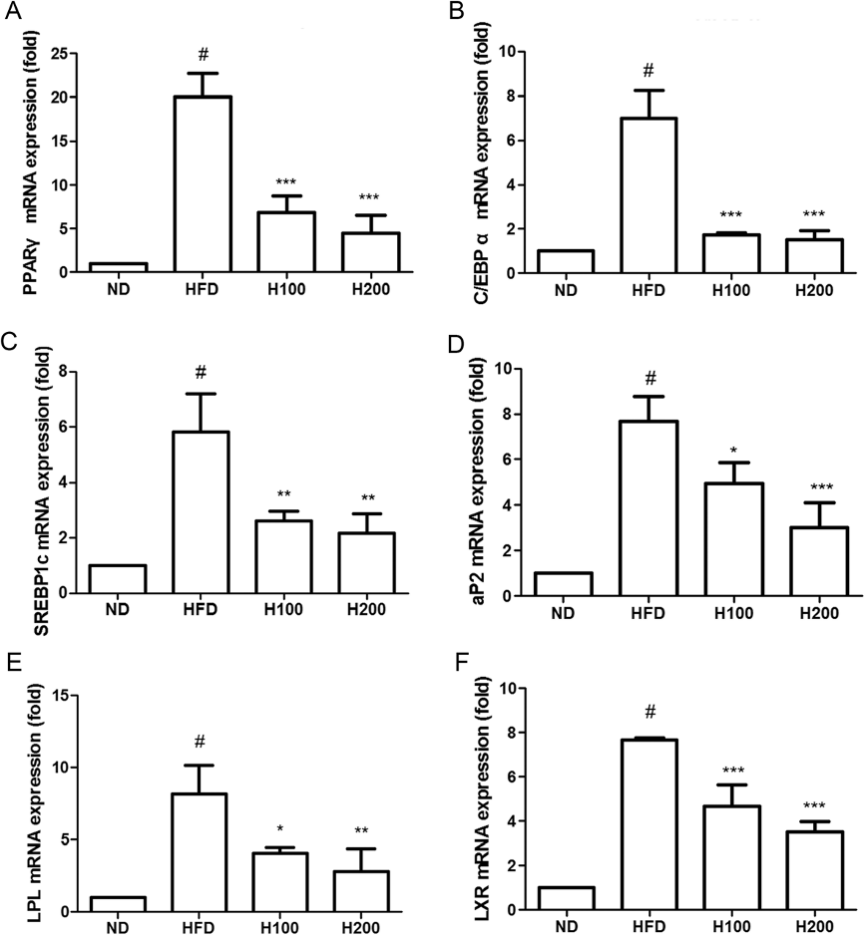
**Effects of HOX-7 on mRNA expression of adipogenesis related genes in epididymal adipose tissue.** mRNA expression of **(A)** PPARγ, **(B)** C/EBPα, **(C)** SREBP1c, **(D)** aP2, **(E)** LPL, **(F)** LXR. Total RNA was prepared for the real-time-PCR analysis of adipogenesis-related genes expressions from adipose tissues. Real-time PCR analysis was conducted using a Step One Plus® Real-time PCR system. Data were normalized to the GAPDH mRNA levels. ND: Normal diet group; HFD: High fat diet group; H100: HOX-7 (100 mg/kg/day, po) treated with HFD group; H200: HOX-7 (200 mg/kg/day, po) treated with HFD group. The values are represented as mean ± SE (n = 8) of three independent experiments. ^#^ *P* < 0.05 versus ND group. *P < 0.05, **P < 0.01, and ***P < 0.001 versus HFD group.

To determine the inhibitory effects of HOX-7 on adipogenic mRNA expression induced by the HFD in epididymal adipose tissue, we carried out a quantitative real-time PCR analysis. HOX-7 decreased the expression of mRNA with regard to adipogenesis. PPARγ, the adipogenic key transcription factor, was significantly reduced in the adipose tissue taken from the HOX-7-treated HFD groups in a concentration dependent manner (Figure 3A). In addition, C/EBPα and SREBP1c showed similarly reduced expression (Figure 3B and C). Other adipogenic markers such as adipocyte P2 (aP2), lipoprotein lipase (LPL), and liver X receptor (LXR) were markedly reduced in the epididymal adipose tissue of HOX-7-treated mice (Figure 3D - F).

### Effects of HOX-7 on the expression of adipocyte-specific proteins

**Figure 4:**
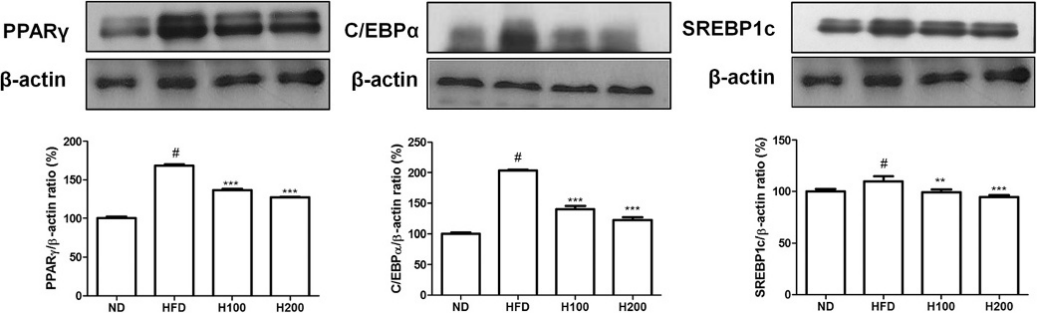
**Effects of HOX-7 on expression of adipogenesis related proteins.** Protein levels of PPARγ, C/EBPα, and SREBP1c were determined by Western blotting. ND: Normal diet group; HFD: High fat diet group; H100: HOX-7 (100 mg/kg/day, po) treated with HFD group; H200: HOX-7 (200 mg/kg/day, po) treated with HFD group. The values are represented as mean ± SE of three independent experiments. ^#^ *P* < 0.05 versus ND group. **P < 0.01, and ***P < 0.001 versus HFD group.

To confirm the inhibitory effects of HOX-7 on adipogenic mRNA expression, the expression of key adipogenic proteins was investigated by Western blot analysis. In parallel with mRNA expression, the amounts of the PPARγ, C/EBPα, and SREBP1c proteins were reduced in epididymal adipose tissue obtained from HOX-7-treated HFD mice than from mice given HFD alone (Figure 4).

## Discussion

Globally, obesity is the most prevalent health problem affecting all age groups and numerous studies have focused on developing natural anti-obesity herbal medicines [23]. In line with these worldwide trends in anti-obesity research, we investigated the anti-obesity effect of HOX-7 and showed that it significantly reduced body and visceral fat weight in HFD-induced obese mouse model. In addition, we found that HOX-7 suppressed adipogenesis in epididymal adipose tissues.

Obesity is the result of a complicated process regulated by a number of adipogenic transcription factors [24]. PPARγ is a ligand-activated transcriptional factor that has a key role in adipocyte differentiation [25]. It is mostly expressed in adipose tissues and required for differentiation of preadipocytes to mature adipocytes [25]. PPARγ activity is evoked when adipocyte differentiation starts, and it transfers hormonal stimulation to its target genes, one of which is C/EBPα. When C/EBPα responds to a PPARγ stimulus, it cooperates with PPARγ to achieve adipocyte differentiation. Although the initial expression of C/EBPα is induced by the PPARγ signal, it functions to further strengthen PPARγ activities by stimulating many specific genes required for adipocyte differentiation [26]. Additionally, SREBP1c is required for optimal adipocyte differentiation. It regulates the transcription of many genes related to lipid, fatty acid, and glucose metabolism [27]. Interestingly, SREBP1c participates in the production of an endogenous PPARγ ligand that reinforces PPARγ activity, which means that PPARγ might be a target gene of SREBP1c [28]. Thus, PPARγ, C/EBPα, and SREBP1c are believed to be crucial to, and perform a leading role in, the progression of adipocyte differentiation. In this study, we examined the effect of HOX-7 on the expression of various adipogenesis-related genes and found that HOX-7 inhibited the expression of PPARγ, C/EBPα, and SREBP1c in epididymal adipose tissues. These results suggest that HOX-7 exerts anti-obesity effects through the regulation of adipogenic transcription factors.

The transcription factors aP2 and LPL are downstream targets of PPARγ and C/EBPα [29]. Therefore, the gene expression of aP2 and LPL indicates the transcriptional activities of PPARγ and C/EBPα. We found that HOX-7 suppressed the gene expression of aP2 and LPL, thus further confirming the inhibitory effect of HOX-7 on the expression of PPARγ and C/EBPα. LXR is primarily considered a regulator of cholesterol and fatty acid metabolism in liver tissue and macrophages [30]. LXR similarly stimulates adipocyte differentiation through the induction of PPARγ expression [31]. LXR forms a positive relationship with PPARγ and mediates adipogenesis by affecting adipogenic transcriptional factors. In this study, HOX-7 also attenuated the expression of LXR at the mRNA level.

## Conclusions

In summary, HOX-7 significantly suppressed body and visceral fat weight in an HFD-induced obese mouse model. HOX-7 inhibited the expression of adipogenesis-related genes, such as PPARγ, C/EBPα, and SREBP1c. Our findings suggest that HOX-7 could represent a novel natural anti-obesity herbal medicine.
